# The genetics of neurodegenerative diseases is the genetics of age-related damage clearance failure

**DOI:** 10.1038/s41380-025-02911-7

**Published:** 2025-01-29

**Authors:** John Hardy, Valentina Escott-Price

**Affiliations:** 1 Department of Neurodegenerative Disease, UCL Institute of Neurology, United Kingdom and UK Dementia Research Institute at UCL, London, UK; 2 Department of Psychological Medicine and Clinical Neuroscience, Cardiff University, United Kingdom and UK Dementia Research Institute at Cardiff, Cardiff University, Cardiff, UK

**Keywords:** Diseases, Genetics, Psychiatric disorders

## Abstract

In this perspective we draw together the data from the genome wide association studies for Alzheimer’s disease, Parkinson’s disease and the tauopathies and reach the conclusion that in each case, most of the risk loci are involved in the clearance of the deposited proteins: in Alzheimer’s disease, the microglial removal of Aβ, in the synucleinopathies, the lysosomal clearance of synuclein and in the tauopathies, the removal of tau protein by the ubiquitin proteasome. We make the point that most loci identified through genome wide association studies are not strictly pathogenic but rather relate to failures to remove age related damage. We discuss these issues in the context of copathologies in elderly individuals and the prediction of disease through polygenic risk score analysis at different ages. Finally, we discuss what analytic approaches are needed now that we have adequately sized case control analyses in white populations.

## Background

The analysis of late onset neurodegenerative diseases through genome wide association studies and whole exome and whole genome sequencing has been extremely successful in terms of finding variants which increase the risk of these diseases [1]. These loci are often described as pathogenic (Oxford English Dictionary: “capable of causing disease”). Most of the loci which have been described, however, are in damage response pathways and are loss of, or reduced function, alleles: in Alzheimer’s disease, they are usually microglial and involved in lipid metabolism [2], in Parkinson’s disease they are often lysosomal or involved in mitophagy [3] and in tangle diseases, they seem to be involved in the ubiquitin proteasome system (although in this case, the data is less certain because the number of cases underlying the Genome-Wide association studies (GWAS) studies are lower) [4]. Thus, a general principle is that variants which increase the risk of disease are reduced function variants in the pathways involved in the removal of damaged proteins and other cellular components [4]. The diseases in general, are therefore, the results of age-related failures in damage clearance. While the removal pathways outlined above (microglia, lysosomes, ubiquitin proteasome) are broadly distinguishable, they are clearly linked and not completely separable [5–7]. In this context, therefore, the term “pathogenic” is misleading since they are variants which are less good at stopping disease rather than variants causing disease.

With this background, one can start to interpret disease associated loci and perhaps to resolve some of the outstanding questions about the pathogeneses of these diseases.

1. What underlies the specific protein depositions which occur in the diseases?
2. Why are co-pathologies very common in individuals with late onset disease?
3. Why are some loci associated with differing pathologies?
4. What underlies the observation of incomplete penetrance in near mendelian loci for neurodegenerative diseases?
5. Why are these diseases, age dependent?

In this context, with a more holistic view of disease pathogenesis, we can discuss disease prediction in these age-related diseases where allele frequencies in the population are influenced by age. This will be useful as we try and develop therapies for these complex diseases with mixed pathologies.

### What underlies the specific protein depositions?

The deposited proteins, Aβ (from *APP*), synuclein and tau are all derived from highly expressed proteins which are close to their deposition thresholds. This has been determined both from protein chemistry work [8] and from the genetic observations that the major neurodegenerative diseases can all be caused by gene duplications for the deposited proteins: *APP*, *SNCA*, *MAPT* [9–11] and that normal genetic variability at these loci contribute to disease risk [12, 13]. We now realise that this variability may be direct, through promoter or splice site variability, or more indirect through influencing the expression of antisense transcripts or “pseudogenes” which can act as dummy ligands. These observations *in toto* clearly show the amount of synthesis of the deposited protein is one key factor in determining risk. Work in vivo in Alzheimer’s disease, using non-radioactive isotopic labelling has shown that in presenilin mutation carriers the predominant problem relates to Aβ production consistent with this view [14]. Genetic analysis of late onset sporadic diseases has, however, suggested that genetic variability in the production of the proteins is not the major determinant of risk but rather that most of the loci identified in the analyses of these forms of the diseases relate to the protein clearance pathways with risk variants, in general being those which reduce flux through the relevant clearance pathways. Again, in Alzheimer’s disease this suggestion is consistent with in vivo data suggesting late onset AD cases have reduced clearance of Aβ [15].

To summarise, all the genetic data for these diseases are consistent with the view that in the protein deposition disorders, the major determinant of risk is the balance between production of the deposited protein and its clearance: factors which increase production or factors which reduce clearance increase that risk. Furthermore, the capacity of the clearance pathways show age related declines [16, 17] and this age related decline in clearance capacity may be the underlying reason for these diseases being of late onset.

### Why are co-pathologies very common in individuals with late onset disease?

Neuropathologists and epidemiologists often point to the fact that, especially in the elderly, detailed brain examination always shows the presence of multiple pathologies [18]. Alzheimer’s disease diagnosis requires the presence of amyloid plaques and tau tangles, but often additionally has some Lewy body pathology. Dementia with Lewy bodies is defined by the presence of synuclein containing Lewy bodies, sometimes as the sole pathology but often in the presence of amyloid plaques and sometimes with some tau tangles too. These co-pathologies also occur in other diseases: for example, Parkinson Dementia Complex of Guam is classically a tau tangle disease but frequently also has synuclein Lewy body pathology [19]. With all the protein deposition disorders, there is also typically a contribution of vascular pathology [20]. This occurrence of multiple pathologies is clearly mechanistically important and has implications for therapeutic strategies since these are usually aimed at one pathology. What has not been determined is the extent to which the pathologies are dependent upon each other or coincidental. This is difficult to assess because of the possibility that ascertainment bias leads to the identification of brains with multiple pathogies which might all contribute independently to the clinical picture [21, 22]. No direct links have been shown between any two pathologies, although transgenic mouse work has suggested that amyloid pathology can potentiate both tau [23] and synuclein pathology [24].

Attempts to link the pathologies have generally focused on the idea that one pathology is upstream of the other (e.g. [25]) although attempts to clearly link the different pathologies have not generally been fruitful. The suggestion that each pathology largely represents a failing protein clearance pathway:- plaques: microglia, Lewy bodies: lysosomes, tangles: the ubiquitin proteasome, suggests an alternative relationship between the pathologies which is that spillover from one clearance pathway to the others, causes their failure too. As support for this notion is the observation that tangle formation is almost universal in the medial temporal lobe and some subcortical structures in the elderly (termed Primary Age Related Tauopathy: PART) [26]. Our interpretation of this observation is that in these elderly individuals the tau clearance pathway is failing in these cells. In such a context the small additional load caused by cortical amyloid deposition could cause spread of tangle pathology to the cortex. In other words, one does not have to postulate a direct relationship between the pathologies, but rather that failing inter-related clearance pathways leads to spillover to other pathways causing them to fail too.

### Why are some loci associated with differing pathologies?

The observation that some genetic loci could be associated with completely different pathologies or anatomies has been a surprise, although the fact *APP* and *PSEN* mutations could both lead to amyloid pathology with a mixture of tangle and Lewy body pathology has long been appreciated [27, 28]. That occurrence of the same *LRRK2* mutations can lead to the different pathologies, Lewy bodies or tangles, with the same clinical features (Parkinson’s disease) in both cases [29] brought the paradox of differing outcomes to the fore: the mutation could be the same and the clinical picture seemed to be identical (suggesting the same neurons were affected) and yet the histopathology was different. This paradox is difficult to reconcile with the view that the mutations actively “cause” the pathology but is easier to reconcile with the view that the mutation leads to an inadequate response to damage. In this scenario, the mutant allele exposes a failure in damage response but the precise pathology will depend on the type of damage. Thus, in this example, *LRRK2* would be predicted to encode a protein at a point of integration in different damage repair pathways. Another similar example is the pathogenesis of Frontotemporal dementia (FTD) and Motor Neurone disease (MND). Both these clinical syndromes can be caused by point mutations in either *VCP* or *SQSTM* or by the *C9orf72* expansion [30].

### What underlies the observation of incomplete penetrance in near mendelian loci for neurodegenerative diseases?

**Fig. 1:**
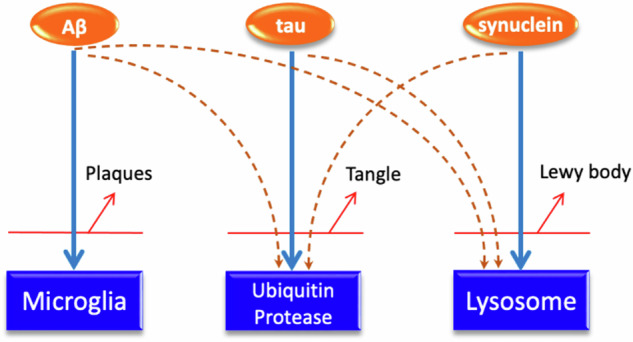
Cartoon suggesting the possible relationships between the different disease pathologies: amyloid, cleared largely by the microglia: tau, clearly largely by the ubiquitin proteasome and synuclein, cleared mainy through the lysosome. However, these clearance pathways are not mutually exclusive, and as one fails, it overloads the other pathways pushing them to fail and cause their substrates to build up. Thus, the different pathologes need not have direct connections, but rather be indirectly connected by clearance failure.

If disease loci are generally components of damage response and clearance mechanisms with the deposited proteins revealing the precise failing mechanism, then the occurrence of pathology will depend on the age dependent capacity of the relevant clearance system and how much other damage the is pathway dealing with [17]. This last factor may be age related and environment-induced damage may include spillover damage from other overloaded pathways (Fig. 1). This, rather complex model of pathogenesis, allows for the role of environmental factors in disease initiation and suggests that incomplete penetrance may in part at least reflect both genetic and environmental components.

### Why are these diseases, age dependent?

It has always been a puzzle as to why these diseases occur in middle-aged to elderly individuals when the underlying genetic architecture is present from birth but, in the context of failing damage response systems, this clearly becomes easier to understand [17]. As age related damage accrues, then clearance systems which were adequate early in life, become overwhelmed.

## Disease manifestation in the context of failing damage clearance and age dependent allele frequencies

**Fig. 2:**
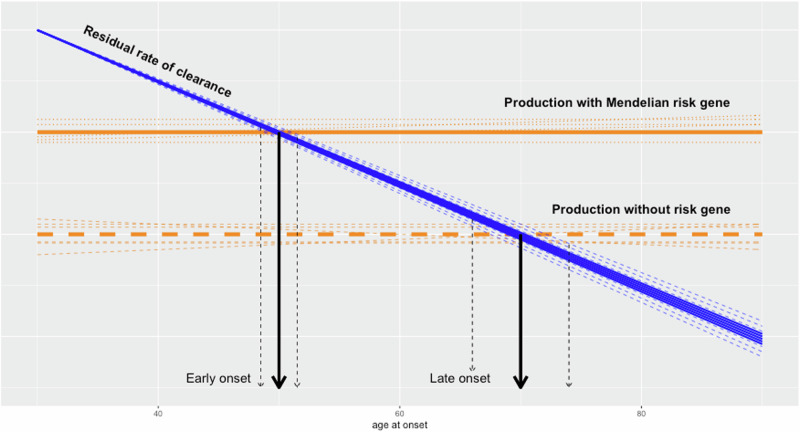
Cartoon showing the reduction of clearance capacity by age (blue line), with the shading arpund the line suggesting variability. Horizontal (orange lines) showing rate of production with shading around the line suggesting variability. Vertical (black lines) marking the intercept when production exceeds clearance and deposition starts.

Once we start to envision disease risk being a property of failing interrelated homeostatic damage clearance systems during ageing, it becomes clear both that mixed pathologies are almost inevitable and that an individual’s pathology and precise risk will be influenced by the rate of decline in the relevant clearance capacity, as well as by the relevant allele effect sizes at that age. Since these neurodegenerative diseases, especially Alzheimer’s disease, are major causes of mortality, the risk of pathology at any age will vary dependent on the clearance capacity at that age (Fig. 2). Thus, risk predictions need to incorporate the appropriate allele frequencies for that pathology by age. This means, for example, that risk for dementia will have a different genetic architecture at different ages consistent with the different pathological underpinning of dementia by age [31]. Related to this risk variability, one might expect variable treatment efficacy by age: for example, in the very elderly, where less amyloid pathology may be present and responsible for less of the dementia risk, amyloid removing treatments may have a smaller clinical effect than in those who are younger even though both groups might have the same Alzheimer’s diagnosis.

## How should we proceed with genome wide analyses to both increase diagnostic prediction accuracy and delineate pathogenic pathways?

### Diagnosis

A major lesson to be drawn from the GWAS which have been reported is that for their interpretation, they need to be labelled correctly. Most of the GWAS for “Alzheimer’s disease” have, in fact, been GWAS for dementia [32]. Such GWAS may have some value in predicting who will develop dementia but it is hazardous to interpret them in terms of pathogenic mechanism (and thus for clinical trial inclusion) since they are likely to include cases with different underlying pathologies. Ideally, one would like to carry out GWAS of pathologically confirmed cases and use these as the basis for prediction. However, the case series available for these types of pathology confirmed GWAS are small. Because of this, restricted analyses of loci implicated in the clinically based GWAS are a partial substitute. The value of complementing these clinical and pathological case series, with analyses of biomarker defined phenotypes is that, although these biomarker-defined analyses are too small for genome wide studies, their utility is that they break the pathogenesis of disease into component parts as exemplified by the GWAS of amyloid PET positivity has shown [33] (see below).

### Age specific analyses

As discussed above, there are two age dependent variables which complicate the application of polygenic risk score at different ages. The first is that the pathologic substrates of diseases changes by age with more mixed pathology at later ages [34, 35]. This changing landscape of pathology by age will depend, at least in part, on the different age dependencies of damage.

This will include mitochondrial damage [36] and oxidative stress [37] leading to mitophagy [36, 38], membrane and white matter damage [39, 40] leading to inflammation and microglial activation [41] and autophagy [42].

**Fig. 3:**
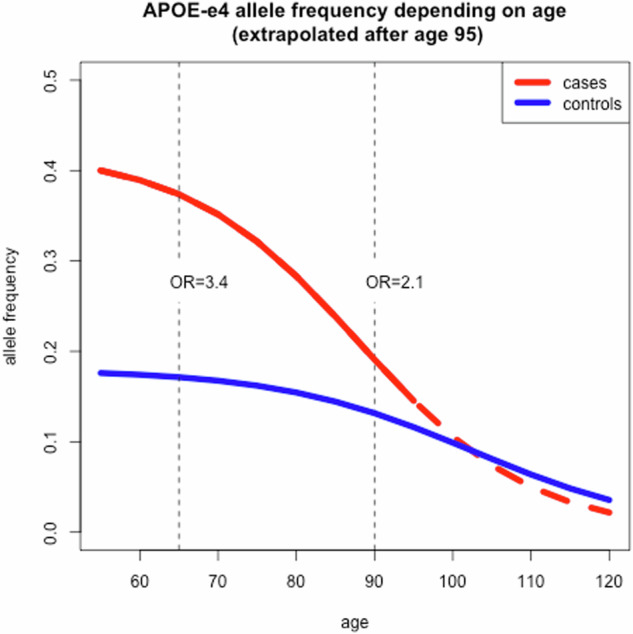
APOE4 allele frequency by age in cases (red) and controls (blue) showing that the odds ratio between cases and controls alters by age. Since APOE4 has the largest effect on Alzheimer risk, it is likely that this allele shows the largest interaction with age but other risk alleles are likely to have the same type of age dependent effect.

For us to maximise the utility of polygenic risk scores in the context of this changing pattern of damage we will need to develop GWAS analyses stratified by age with cases and controls being adequately age matched. Most of the published GWAS for neurodegenerative disorders are not age matched and thus are unsuitable as substrates for these analyses. The second issue is that the background allele frequencies change because of allele specific mortalities by age; this is especially true at the APOE locus [43–45] but is also true of others [46, 47] (Fig. 3).

## Studies in Non-European populations

Nearly all the published studies of the genetic wide analyses of neurodegenerative diseases have been carried out in Northern European cohorts. This is unsatisfactory for three reasons: firstly, it means that any therapies which are based upon genetic knowledge will be incompletely available to other populations, secondly as we identify more variants associated with disease in diverse populations it will give us deeper insights into mechanisms, and thirdly, the different haplotype structures mean that precise localisation of pathogenic loci is facilitated by cross ethnic comparisons. In the last period, some progress in the genome wide analyses has begun to be made with GWAS for both Alzheimer’s disease and Parkinson’s disease being published in Asian and African cohorts [48–51]. These analyses have shown, in general, that the same loci are involved in disease across ethnic groups, but that the weights and precise and mechanisms variants at those loci are different. As examples of this the *ABCA7* internally deleted allele in African American samples is associated with an increased risk of Alzheimer’s disease in such populations [52] and the prevalent *GBAP* allele associated with PD risk in African derived samples [51] are both examples where the profiles of risk and the precise disease mechanisms are different in different populations.

Genetic risk differences between populations occur for three main reasons, (a) allele frequency variations, (b) linkage disequilibrium structure, and (c) disease risk effect sizes caused by gene x environment interactions. Therefore, to develop interventions and theatments, with focus shifted from individual genes to the underlying biological pathways they are compenents of.

## The road ahead

Genome wide studies have revolutionised our views of the underlying biology of neurodegenerative disease and there has naturally been an inclination to continue to increase the size of such studies since larger studies inevitably identify more loci. We would argue that rather than simply increasing the sizes of such studies, resources would be better allocated to carrying out more focused analyses. These should include analyses of quantitative biomarkers and pathology and of rates of disease progression. Our research, along with others’, shows that variations in CSF and plasma biomarkers—specifically free Aβ peptides (chain lengths 40 and 42), glial fibrillary acidic protein (GFAP), neurofilament light (NFL), and p-Tau 181 and 217—are not fully captured by genetic factors, and can enhance disease risk prediction accuracy [53]. All of these biomarkers, however, were also associated with age at the time of sample collection, suggesting that they are sensitive to age or to preclinical age-related neurodegenerative pathologies. Therefore, as is the case for genetic risk prediction, age should be factored in when interpreting disease risk using biomarkers.

### Pathology confirmed GWAS

By examining the genetcs of different components of the disease pathology we should be able to derive a more fine grained dissection of their pathogenetic mechanisms. For example, we have shown that amyloid deposition and the occurrence of dementia involve distinct biological processes: the former being almost exclusively apoe dependent and the latter dependent on the microglial response [33]. More recently, a pathology based study [54] found that genes associated with specific neuropathology endophenotypes often concurred with previous GWAS of neurodegenerative diseases: thus amyloid plaques and tangle numbers were apoe dependent, tdp43 pathology was associated with the FTD genes GRN and TMEM106B and vascular pathology showed an association with the stroke locus COL4A1 [55].

Clearly therefore, these genetic findings go some way to aligning with the observations of frequent co-pathologies in the elderly. In addition to these previously described loci, however, there are many entirely new loci which may relate directly to the occurrence of copathology since a key point is whether pathologies are dependent or independent. To understand mixed dementia, the outcome in the pathology confirmed GWAS should develop from the assessment of specific neuropathologies to endophenotype to clusters of such co-pathologies. Such analyses would not attempt to prove genome wide (10^−8^) significance since numbers will inevitably be small but rather would test the GWAS from clinical samples as candidate genes to determine which loci were genuinely associated with “mixed” disease pathogenesis.

#### GWAS of age specific risks of disease

Again, such analyses would not require the proof of genome wide significance. These analyses will be needed for developing polygenic risk analysis predictions to aid with early diagnoses at different ages.

#### GWAS of imaging and fluid biomarkers and of disease related phenotypes, and of age at onset and rate of decline in the disease

Such analyses have the potential to dissect the pathways to pathogenesis as two examples illustrate (a) the GWAS of amyloid positivity compared to the GWAS of pathologically confirmed Alzheimer’s disease broke Alzheimer pathogenesis into amyloid deposition and then dementia in that context (b) the comparison of the GWAS for the rapid eye movement (REM) behaviour disorder, Parkinson’s disease and Dementia with Lewy bodies shows many overlapping loci as well as many loci with different effects allowing the discrimination of different elements of the pathogenic cascade.

## Conclusions

To a surprising degree, GWAs have revealed not only the genetic risks and pathways underlying neurodegenerative disease and their relationships to the ageing process, they are also illuminating the unappreciated mechanisms of gene regulation. By moving beyond case control analyses and by comparing biomarker GWAS with disease GWAS and contrasting GWAS of related phenotypes with each other we are beginning to dissect different stages of disease pathogenesis: through the comparison of GWAS in different populations we are getting a greater window into the richness and complexity of genetic mechanisms. There is still much to be done: improving genetic prediction in non-white populations and investigating whether epistatic interactions explain missing heritability are two examples where progress is needed.
